# Using Weakly Conserved Motifs Hidden in Secretion Signals to Identify Type-III Effectors from Bacterial Pathogen Genomes

**DOI:** 10.1371/journal.pone.0056632

**Published:** 2013-02-20

**Authors:** Xiaobao Dong, Yong-Jun Zhang, Ziding Zhang

**Affiliations:** 1 State Key Laboratory of Agrobiotechnology, College of Biological Sciences, China Agricultural University, Beijing, China; 2 State Key Laboratory for Biology of Plant Diseases and Insect Pests, Institute of Plant Protection, Chinese Academy of Agricultural Sciences, Beijing, China; University of Alberta, Canada

## Abstract

**Background:**

As one of the most important virulence factor types in gram-negative pathogenic bacteria, type-III effectors (TTEs) play a crucial role in pathogen-host interactions by directly influencing immune signaling pathways within host cells. Based on the hypothesis that type-III secretion signals may be comprised of some weakly conserved sequence motifs, here we used profile-based amino acid pair information to develop an accurate TTE predictor.

**Results:**

For a TTE or non-TTE, we first used a hidden Markov model-based sequence searching method (i.e., HHblits) to detect its weakly homologous sequences and extracted the profile-based *k*-spaced amino acid pair composition (HH-CKSAAP) from the N-terminal sequences. In the next step, the feature vector HH-CKSAAP was used to train a linear support vector machine model, which we designate as BEAN (Bacterial Effector ANalyzer). We compared our method with four existing TTE predictors through an independent test set, and our method revealed improved performance. Furthermore, we listed the most predictive amino acid pairs according to their weights in the established classification model. Evolutionary analysis shows that predictive amino acid pairs tend to be more conserved. Some predictive amino acid pairs also show significantly different position distributions between TTEs and non-TTEs. These analyses confirmed that some weakly conserved sequence motifs may play important roles in type-III secretion signals. Finally, we also used BEAN to scan one plant pathogen genome and showed that BEAN can be used for genome-wide TTE identification. The webserver and stand-alone version of BEAN are available at http://protein.cau.edu.cn:8080/bean/.

## Introduction

After having coevolved with their hosts for hundreds of millions of years, gram-negative pathogenic bacteria acquired a specific type of proteins known as type-III effectors (TTEs), which are able to suppress host immunity through mimicking host functional proteins, modifying components in the immune signal pathway or even directly regulating host gene expression [1], [2]. As a result, the host range of one pathogenic bacterium is greatly influenced by its repertoires of TTEs [3], [4].

Elaborate experimental strategies, such as translocation assays of labeled TTE candidates [5]–[7] and functional screening for TTEs based on hypersensitive response (HR) in plants [8], have been designed to identify TTEs from pathogen genomes. Thanks to community-wide efforts over many years, hundreds of TTEs have been identified from model gram-negative pathogenic organisms such as *Escherichia coli*, *Salmonella enterica*, and *Pseudomonas syringae* [9]. Even so, it is still an arduous and time-consuming task to conduct these experimental approaches on the whole pathogen genome. Therefore, the computational identification of TTEs is highly desired, and it is playing an increasingly important role in accelerating the identification of TTEs from newly sequenced pathogen genomes.

The fast evolutionary rate of TTEs impedes the use of traditional bioinformatics methods such as sequence similarity searches to correctly identify TTEs from newly sequenced pathogen genomes. Since 2009, a series of state-of-the-art machine learning-based TTE predictors have been developed [10]–[15]. Typical methods include the naïve Bayes algorithm EffectiveT3 [14], two support vector machine (SVM) predictors (SIEVE [15] using both protein and DNA information and BPBAac [10] using a sequence encoding method called bi-profile), and an method based on artificial neural network (ANN) [12]. Generally, existing machine learning methods are still impractical with a high false positive rate (FPR) when they are used on a whole bacterial genome [16]. Recently, a meta-approach has also been proposed to predict TTEs from the genome level [16]. However, this meta-approach would be infeasible for some newly sequenced bacterial genomes, in which the gene expression data are not available.

Pathogenic bacteria inject TTEs into host cells using a complicated molecular machine called a type-III secretion system (T3SS). Although our current knowledge of type-III secretion signals is still very limited, some type-III secretion signal-related features have been observed. Previous studies have indicated that the first 20–30 amino acids in the N-terminal sequences of TTEs were enough to target them into host cells [17], [18]. It has also been proposed that the secondary structure of mRNA, which encodes TTEs, could be the carrier of secretion signals [19]. Other researchers have reported that some TTEs’ secretion processes require the participation of chaperones [20]. Moreover, a statistical analysis between TTEs and non-TTEs also showed different residue propensities and structural properties in the N-terminal sequences [14], [16], [21]. For instance, polar amino acids such as serine and threonine are enriched, but hydrophobic and acidic amino acids are depleted within the first 30 residues of TTEs. Intrinsic disorder is also deemed to be a possible universal characteristic for type-III secretion signals [22]. Coding sequence analysis shows unusual G+C content and codon usage bias in TTEs [16], implying that one pathogen can acquire TTEs from other pathogens via horizontal gene transfer. Although none of the above features can be used individually to effectively discriminate TTEs from other proteins in bacterial genomes, these observations have clearly suggested that type-III secretion signals should be very diverse.

Sequence motifs are evolutionarily plastic sequence fragments that have been reported to mediate protein-protein interaction and be enriched in intrinsically disordered regions of proteins [23], [24]. In eukaryotic cells, they can target proteins to specific cellular compartments [25]. In the type-II and type-IV *sec*-dependent secretion pathways of gram-negative bacteria, secretion signals have a motif-like amino acid composition within the N-terminal of protein sequences [26]. Although the concept of sequence motif-related type-III secretion signals has been proposed [15], it is still not widely accepted, and the experimental verification of motifs in TTEs is very limited. In this work, we further hypothesize that specific sequence motifs that mediate the interaction between TTE and T3SS should play important roles in type-III secretion signals, and we can identify TTEs using this sequence motif-related information. Based on this hypothesis, we explored the use of *k*-spaced amino acid pair information to predict TTEs, as *k*-spaced amino acid pairs could be regarded as the basic elements of short sequence motifs. In fact, the composition of *k*-spaced amino acid pairs (CKSAAP) has become a useful feature construction of a sequence or a sequence fragment, which has been successfully employed for diverse bioinformatics tasks, including the prediction of protein flexible/rigid regions [27], protein crystallization [28], protein structural classes [29], membrane protein types [30], O-glycosylation sites [31], palmitoylation sites [32] and ubiquitination sites [33].

**Figure 1:**
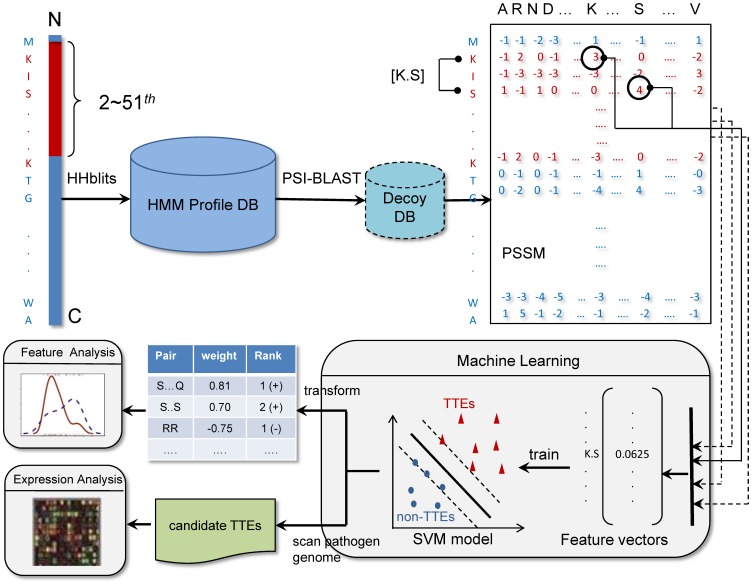
Overview of the proposed TTE predictor BEAN. A full-length sequence is used to construct its profile (PSSM) via HHblits search. Only the first 2–51 residues of the N-terminal are used to compute the profile-based *k*-spaced amino acid pair composition. Then, the feature vectors with a dimensionality of 1600 are taken as input to train a linear SVM classification model. Through the parameter transformation of the established model, we obtained the weights of each *k*-spaced amino acid pair and analyzed the evolutionary conservation and sequence position distribution of each pair. We also used our BEAN to scan a pathogen genome and identify TTE candidates.

We called our method BEAN (Bacterial Effector ANalyzer). In our method, the profile of a TTE or non-TTE sequence was first constructed based on a hidden Markov model (HMM) searching strategy. Then, we used CKSAAP in the N-terminal sequences extracted from the resulting profiles as input to train a linear SVM model (Figure 1). We characterized the performance of BEAN through 5-fold cross validation tests, and we also benchmarked BEAN against four existing TTE predictors based on an independent test set. We applied BEAN to conduct a genome-wide TTE prediction in one plant pathogen, *Ralstonia solanacearum* GMI1000. More importantly, we discussed T3SS-related motifs through the evolutionary analysis and position distribution analysis of the most predictive amino acid pairs in TTEs. It is hoped that the current work can provide some new information regarding the secretion signal in TTEs.

## Materials and Methods

### Data Collection and Preprocessing

To train our classification model, we primarily used a non-redundant dataset compiled by Wang et al. (2011) [10], which contains 154 TTEs collected from the literature and 308 non-TTEs randomly sampled from pathogen proteomes [10].We used these 462 sequences to train our prediction model and construct our webserver. To investigate the influence of negative samples, we also collected eight well-studied gram-negative bacterial proteomes (*Escherichia coli* O157:H7, *Salmonella enterica* serovar Typhimurium, *Pseudomonas syringae* DC3000, *Yesinia pestis* bv. Antiqua, *Chlamydia trachomatics*, *Shigella flexneri*, *Yesinia enteroclitica*, and *Burkholderia pesudomallei* ) from Uniprot [34]. We only retained sequences satisfying the following criteria: i) they should have been reviewed in Uniprot; ii) there are no words matching “T3SS effector” in the description section or the regular expression “/Secreted.*type.*(III|three) secretion system/” in the subcellular location section of the corresponding Uniprot records; and iii) they should share a <95% sequence identity with known TTEs. We obtained 7143 sequences that were regarded as negative samples (i.e., non-TTEs). The sequence redundancy of these 7143 non-TTEs was further removed using Arnold et al.’s method [14]. In brief, these non-TTEs were grouped according to their sequence similarity scores generated from Jaligner (http://jaligner.sourceforge.net). If any two sequences were assigned a similarity bit score 0.15 times larger than the self-to-self similarity bit score from any of these two sequences, they were grouped as one. For each group, only one randomly selected sequence was kept. Finally, four non-TTE sets were randomly sampled and the size of each set is equal to 308. We combined Wang et al.’s data and these four non-TTE sets into a dataset called Data1.

To construct an independent test set, 109 newly identified TTEs and 14 experimentally validated non-TTEs were collected manually from literature published after January 2011. Two hundred non-TTE samples were further randomly sampled using the procedures described in the above paragraph. To test the robustness of the performance, the selection of 200 non-TTEs was repeated five times. Finally, we obtained an independent test set (i.e., Data2), which contained 109 TTEs, 14 non-TTEs, and five sets of 200 randomly sampled non-TTEs. Detailed information about Data1 and Data2 is provided in supporting information (Dataset S1 and Dataset S2).

### Homolog Searching and Profile Construction

For each TTE or non-TTE sequence, we used HHblits [35], which implements an improved HMM-HMM profile searching algorithm, to detect its homologs with 2 iterations. Other parameters in HHblits were set to the default values. Note that the HMM profiles of HHblits were built on full-length sequences. To avoid incorrect searching results, the full length sequence of the query was used in this procedure. Then, the resulting sequence homologs plus the query sequence were aligned using the maximum accuracy (MAC) algorithm [36] wrapped in HHsuite to build a multiple sequence alignment (MSA). Subsequently, the obtained MSA was PSI-BLASTed against a decoy NCBI sequence database, which is a very small dummy database created for saving searching time and has been included in HHsuite, to induce PSI-BLAST program to construct a sequence profile, also known as a position specific scoring matrix (PSSM). Due to the coverage limitation of the current HHblits database, some sequences failed to detect any homolog. In these cases, we used PSI-BLAST directly to search the NCBI NR database to construct the query sequence’s PSSM.

### Extracting *k*-spaced Amino Acid Pair Compositions from Profiles

Letting 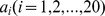 denote one of 20 amino acids, a *k*-spaced amino acid pair could be represented as 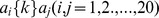, where 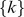 indicates that there are *k* residues between 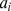 and 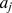 in the original sequence. Specifically, 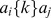 is a dipeptide when *k* is 0. In our work, the maximum *k* was optimally set to 3, meaning that 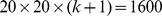 different amino acid pairs were taken into account. Instead of calculating the composition of the 1600 pairs from the original sequence directly, we used a profile-based *k*-spaced amino acid pair composition method [30] to compute the feature vector of each sequence. For a protein sequence with *L* residues, the corresponding PSSM has a dimensionality of 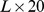. Regarding an amino acid pair 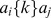, appearing between the position of *m* and 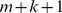, we introduced the following score:
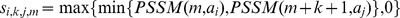
(1)


Where 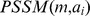 denotes the score of amino acid 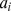 at the 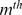 row of PSSM and 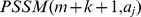 stands for the score of amino acid 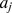 at the 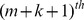 row of PSSM. If 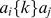 occurs *N* times within a sequence, the composition score of 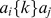 is defined as:
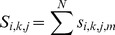
(2)


In this work, the range of N-terminal residues was set to 50 and the first residue in the N-terminal was ignored. That is to say, only the N-terminal residues starting from 2 to 51 were taken into account. Unless otherwise stated, the first N-terminal residue was always ignored in this work. At last, we normalized each feature using the following formula:
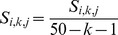
(3)


For simplification purposes, we call the encoding used in this work HH-CKSAAP.

### Training the SVM Model and Extracting the Weights of Amino Acid Pairs

The LIBSVM package (http://www.csie.ntu.edu.tw/~cjlin/libsvm/) was used to train and test our SVM classification model with five-fold cross validation tests. A linear kernel with the parameters cost 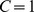 and tolerance of termination criterion 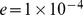 was used to establish the SVM model of BEAN.

For a linear SVM, we want to find the largest decision boundary between two hyperplanes *P_a_* and *P_b_*

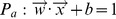
(4)

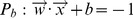
(5)where 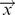 denotes the support vector and 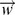 is its corresponding weight vector. Using the dual Lagrange multiplier method, we can obtain the decision hyperplane of linear SVM:
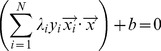
(6)Where 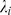 is the Lagrange multiplier, 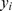 is the class label of the 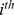 training sample (if it belongs to a TTE 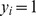, else 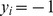), 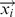 is a feature vector from the training samples, and *N* is the number of training samples. We solve every 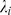 using the training dataset. For a query protein, we take its feature vector 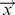 into this formula to decide its class label. We can use the following transformation to obtain the weight of each feature:
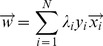
(7)where 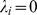 if 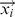 is not a support vector. Since 5-fold cross validation tests based on 154 TTEs and 308 non-TTEs from Wang et al.’s data were conducted in this work, five sets of weighting values for the 1600 features can be calculated. For the purpose of analysis, we only recorded the average weight for each feature.

We further compared BEAN with other non-linear SVM models based on polynomial, sigmoid and Gaussian kernels, respectively. A grid search was used to optimize the parameters of these non-linear models.

### Performance Assessment

We used sensitivity, specificity and the Matthew correlation coefficient (MCC) to evaluate the prediction performance. They are defined as:
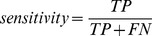
(8)

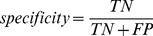
(9)

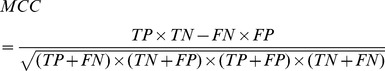
(10)where TP, FP, TN, and FN stand for the number of true positives, the number of false positives, the number of true negatives and the number of false negatives, respectively. The performance was also comprehensively characterized through the receiver operating characteristic (ROC) and precision-recall (PR) curves. The ROC curve plots the true positive rate (i.e., sensitivity) as a function of FPR (i.e., 1-specificity) for all possible thresholds, whereas the PR curve plots precision (i.e., the number of true positives divided by the sum of the true positives and false positives) as a function of recall (i.e., sensitivity).The area under the ROC (auROC) and the area under the PR curve (auPRC) were also calculated. The performance of BEAN was assessed through 5-fold cross validation tests.

### Evolutionary Conservation Analysis of Amino Acid Pairs

We performed an evolutionary analysis of the first 50 residues in 154 TTEs from Data1. Only the analysis of those amino acid pairs with positive weights was carried out. We took the MSAs of the 154 TTEs generated in the profile construction step as the input of Rate4Site [37] to calculate residue conservation scores. We chose empirical Bayesian methods to evaluate the conservation score of each residue. The larger the score is, the less conserved the residue is. In this work, we simply defined the conservation score of one amino acid pair as the larger score of those two amino acids. If there were fewer than five or more than 100 sequences in the MSA of a TTE, we would discard this TTE in our analysis. In the case that one amino acid pair appeared several times in the N-terminal sequence of a TTE, we calculated the average value of all of the corresponding scores. Furthermore, the values in different TTEs were averaged once again.

### Position Distribution Analysis of Amino Acid Pairs

The sequence position distribution of the top 50 positively weighted amino acid pairs in TTEs and non-TTEs was carried out, and only the first 50 N-terminal residues of a protein were taken into account. For an amino acid pair 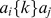 starting from the 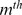 residue in a protein, we defined its sequence position as the average position of the corresponding two amino acids. Since the first amino acid located at the 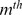 residue and the second amino acid located at the 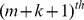 residue of the protein sequence, 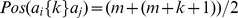. For each amino acid pair, we calculated its occurrence frequency in each possible position and generated the corresponding sequence position distribution using a Gaussian kernel with the default band width of the density function in R (www.r-project.org). Moreover, the average occurrence frequency of these 50 amino acid pairs in each possible position was also counted, and a *loess* function with the default parameters in R was used to plot the trend line of these 50 amino acid pairs’ overall distribution.

## Results

### The Performance of BEAN

**Figure 2:**
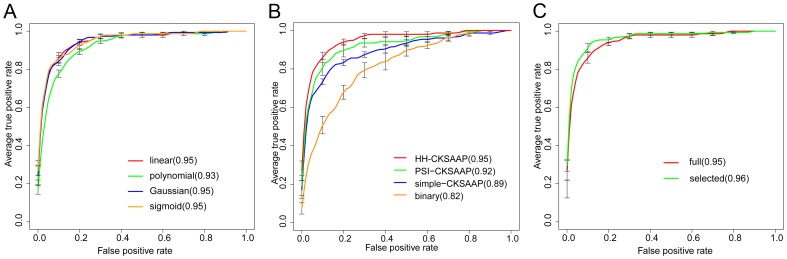
Classification performance of BEAN. (**A**) ROCs of different SVM kernel functions. (**B**) ROCs of different feature extraction methods. (**C**) ROCs of classification models using all 1600 features and the 100 top weighted features. The values in brackets are the auROCs of each model. All of above results are based on Wang’s data.

Through 5-fold cross validation tests on Wang et al.’s data, we systematically tested the prediction performance of BEAN based on different combinations of two parameters related to the input features [i.e., the largest *k*-spaced amino acid pairs (*k_max_*) and the length of N-terminal sequence (*L*)]. Our preliminary parameter optimization showed that the best classification performance corresponds to an MCC value of 0.78 (sensitivity = 78% and specificity = 96%) when *k_max_* is set to 3 and *L* is fixed at 50 and there is no significant improvement of BEAN’s performance when larger *k_max_* and *L* were used. We got similar performance when we trained and tested BEAN with the other four randomly sampled negative datasets in Data1 (Table S1). We also tried three other kernel functions provided by the LIBSVM package, including polynomial, sigmoid and Gaussian kernels. We found that there is no significant performance difference among these kernels (Figure 2A).

We also implemented the other three feature extraction methods, including the binary encoding, the simple composition of *k*-spaced amino acid pairs (simple-CKSAAP) [27], [31], [33] and the PSI-BLAST profile-based composition of *k*-spaced amino acid pairs (PSI-CKSAAP) [30]. Regarding the binary encoding, each amino acid from 50 N-terminal residues is represented as a 20 dimensional binary vector, e.g., A (1,0,0,0,0,0,0,0,0,0,0,0,0,0,0,0,0,0,0,0), C (0,1,0,0,0,0,0,0,0,0,0,0,0,0,0,0,0,0,0,0), etc. Then, the total number of 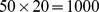 features were used to train a SVM classification model. With respect to simple-CKSAAP, the composition of amino acid pairs is directly extracted from the query sequence. PSI-CKSAAP encoding strategy is very similar to our method. Rather than using HHblits in our method, PSI-CKSAAP only used PSI-BLAST searching to construct the profile. As shown in Figure 2B, the binary encoding, simple-CKSAAP, PSI-CKSAAP and HH-CKSAAP achieved their average auROCs of 0.82, 0.89, 0.92 and 0.95, respectively. We observed all three *k*-spaced amino acids-based encoding methods are much better than the binary encoding. Since the binary encoding is a widely used sequence feature vector, this observation suggested that the *k*-spaced amino acid pairs capture more information related to T3SS signals in comparison to classical sequence features.

To investigate the performance of different types of *k*-spaced amino acid pairs in predicting TTEs, we used each type of *k*-spaced amino acid pairs (*k* = 0, 1, 2 or 3) from simple-CKSAAP to retrain a SVM model and we called the resulting four models as exclusive-CKSAAP. With respect to exclusive-CKSAAP (*k* = 1), for instance, we only considered amino acid pairs 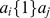 and neglected 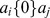, 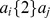 and 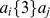. As shown in Figure S1, exclusive-CKSAAP (*k* = 0) (i.e., dipeptide) achieved an accuracy very close to simple-CKSAAP, and the other three exclusive-CKSAAP encoding methods could also yield similarly good performance. In other words, the joint use of different *k*-spaced amino acid pairs (i.e., simple-CKSAAP) could only result in a very limited performance improvement, which could be ascribed to the weak complementary among different types of *k*-spaced amino acid pairs. Thus, the combination of different *k*-spaced amino acid pairs could not effectively increase the signal-to-noise ratio in comparison to each exclusive-CKSAAP model. Even so, the results also clearly suggested that each type of *k*-spaced amino acid pairs contains effective type-III secretion signal information.

We also found both profile-based methods are better than simple-CKSAAP. Comparatively, our method based on HHblits is better than PSI-CKSAAP (Figure 2B). The performance difference implies that the sequence profile information is useful for capturing conserved amino acid pairs and that a more sensitive sequence searching algorithm (e.g., HHblits) can yield a more powerful performance. Taken together, the above performance comparison among different feature extraction methods clearly demonstrated that the success of HH-CKSAAP in predicting TTEs should be ascribed to both of the profile and *k*-spaced amino acid pair information.

### Predictive Amino Acid Pairs

To investigate the amino acid pairs that play dominant roles in classifying TTEs and non-TTEs, we list the pairs having top-ranked weights (i.e., the top 50 positively weighted pairs and the top 50 negatively weighted pairs) in the established SVM model (Table 1). The weight values represent the importance in the decision process of SVM. Generally, the most positively weighted pairs should be enriched in the N-terminal sequences of TTEs and they are informative to discriminate query sequences as positive samples. We observed that over 50% (28/50) of the amino acid pairs in the top 50 positively weighted pairs are comprised of at least one serine. Additionally, the co-located polar and uncharged amino acid pairs also hold a strong majority in these 50 pairs (Table 1). Less than 10% purely hydrophobic amino acid pairs and less than 5% completely charged amino acid pairs were found in the 50 pairs. In contrast, the most negatively weighted pairs are generally depleted in the N-terminal sequences of TTEs, and they should be regarded as useful features for classifying query sequences as non-TTEs. We observed that the top 50 negatively weighted pairs were dominated by hydrophobic amino acid pairs and charged amino acid pairs.

**Table 1:** The top 50 positively and negatively weighted *k*-spaced amino acid pairs.^a^

*Rank*	*Pair(+)*	*Weight*	*Pair(−)*	*Weight*	*Rank*	*Pair(+)*	*Weight*	*Pair(−)*	*Weight*
1	S…Q	0.81	RR	−0.75	26	G.S	0.47	A.I	−0.27
2	S.S	0.70	A.I	−0.61	27	S…T	0.46	PW	−0.27
3	SL	0.68	LI	−0.49	28	I.R	0.46	DY	−0.27
4	P…P	0.64	R.R	−0.42	29	R.E	0.46	Y…G	−0.27
5	LS	0.63	L.L	−0.41	30	V…S	0.45	RP	−0.26
6	G.Q	0.62	C…P	−0.40	31	A.K	0.45	W…C	−0.26
7	S.S	0.62	PK	−0.38	32	P…S	0.45	R.D	−0.26
8	S.N	0.62	R.G	−0.37	33	Q.F	0.45	F…R	−0.25
9	SS	0.62	R…R	−0.36	34	P.S	0.44	R.L	−0.24
10	PS	0.61	LL	−0.36	35	Q.P	0.44	F.L	−0.24
11	S…S	0.60	L.A	−0.36	36	I.S	0.44	V…H	−0.24
12	Q.P	0.57	K.G	−0.35	37	N.Q	0.43	YY	−0.24
13	S…P	0.56	H…Y	−0.35	38	N…S	0.43	T.R	−0.24
14	SN	0.56	AC	−0.34	39	G…P	0.43	F.V	−0.23
15	IQ	0.55	A.F	−0.34	40	S.T	0.42	R…G	−0.23
16	R.G	0.55	Y…R	−0.33	41	QG	0.42	FY	−0.23
17	S.T	0.55	E.E	−0.33	42	A…S	0.42	K.A	−0.23
18	A.S	0.53	I.E	−0.33	43	GP	0.41	E.Q	−0.22
19	S.N	0.53	I.R	−0.31	44	P.G	0.41	E.Y	−0.22
20	NH	0.51	L…I	−0.31	45	S.Q	0.40	F.D	−0.22
21	VA	0.51	LV	−0.30	46	I.K	0.40	MA	−0.22
22	P.P	0.50	AF	−0.29	47	S…G	0.40	V.F	−0.22
23	QT	0.49	T.Y	−0.29	48	S.A	0.40	K.K	−0.22
24	A.S	0.49	L.E	−0.29	49	AS	0.40	K…Y	−0.22
25	T.V	0.47	R.C	−0.28	50	N.F	0.39	RL	−0.22

a “+” indicates a positively weighted pair, “−” denotes a negatively weighted pair, and “.” stands for any amino acid. Of the top-50 positively weighted amino acid pairs, 12, 15, 12 and 11 is from *k* = 0, 1, 2, and 3, respectively. Regarding the top-50 negatively weighted amino acid pairs, the corresponding number of amino acid pairs is 15, 13, 11 and 11, respectively.

The predictive amino acid pairs listed in Table 1 are well consistent with previous studies related to the amino acid propensities in the N-terminal sequences of TTEs and non-TTEs. For instance, Arnold et al. found that serine is the most frequently observed amino acid in the first 25 residues of TTEs, whereas both acidic and alkaline amino acids are depleted in the N-terminal sequences of TTEs in comparison to non-TTEs. Moreover, they also found that amino acid combinations such as acidic-alkaline, polar-polar are informative features in their prediction method [14]. Samudrala et al. also identified some serine-related sequence motifs from the N-terminal sequences of TTEs [15]. Compared with the previous observations, we would like to emphasize that our analysis based on the established linear SVM model placed a greater emphasis on the systematical and comprehensive investigation of the pivotal amino acid pairs from the viewpoint of sequence motifs.

To test the discriminative power of the most predictive amino acid pairs, we only used the 100 amino acid pairs (i.e., the top 50 positively weighted pairs plus the top 50 negatively weighted pairs) to train a linear SVM model. As shown in Figure 2C, the SVM model based on the most predictive features also reveals a very competitive performance.

### Predictive Amino Acid Pairs Tend to be More Conserved in TTEs

**Figure 3:**
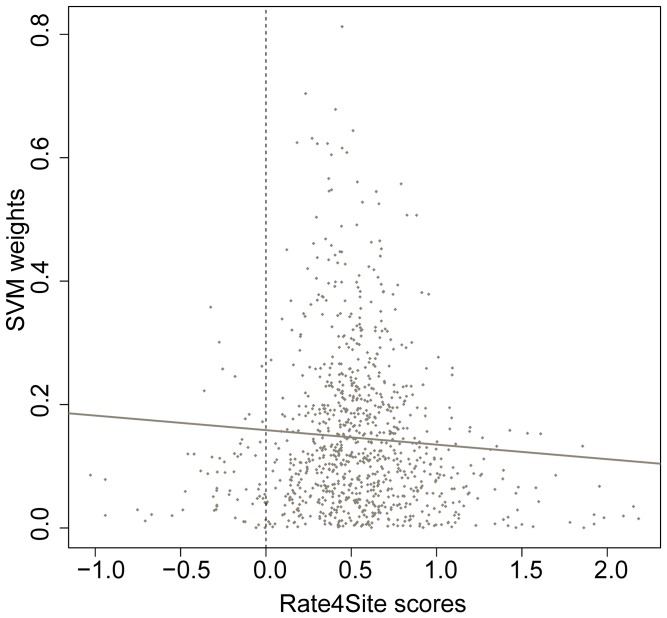
Conservation of positively weighted *k*-spaced amino acid pairs. A lower Rate4Site score corresponds to a more conserved amino acid pair.

To explore the relationship between the conservation of amino acid pairs and their weights, we computed the evolutionary conservation score of every positively weighted *k*-spaced amino acid pair in TTEs. As shown in Figure 3, there is a weak but significant linear correlation between amino acid pairs’ weights and their conservation scores. We found the average Rate4site conservation score of the 50 most predictive amino acid pairs is significantly lower than that of the other amino acid pairs (0.47 vs. 0.54; Mann-Whitney *U*-test, *p* value <0.05 ). Generally, more positively weighted pairs tend to be more conserved, implying that these predictive amino pairs may form some relatively conserved sequence motifs. We also note that most of the amino acid pairs have Rate4Site scores larger than zero (Figure 3), confirming that positive selection is common in the N-terminal residues of TTEs. This mutation-prone selection further explains the difficulty of directly identifying conserved sequence motifs from the MSAs of TTEs.

### Predictive Amino Acid Pairs Show Different Position Distributions between TTEs and non-TTEs

**Figure 4:**
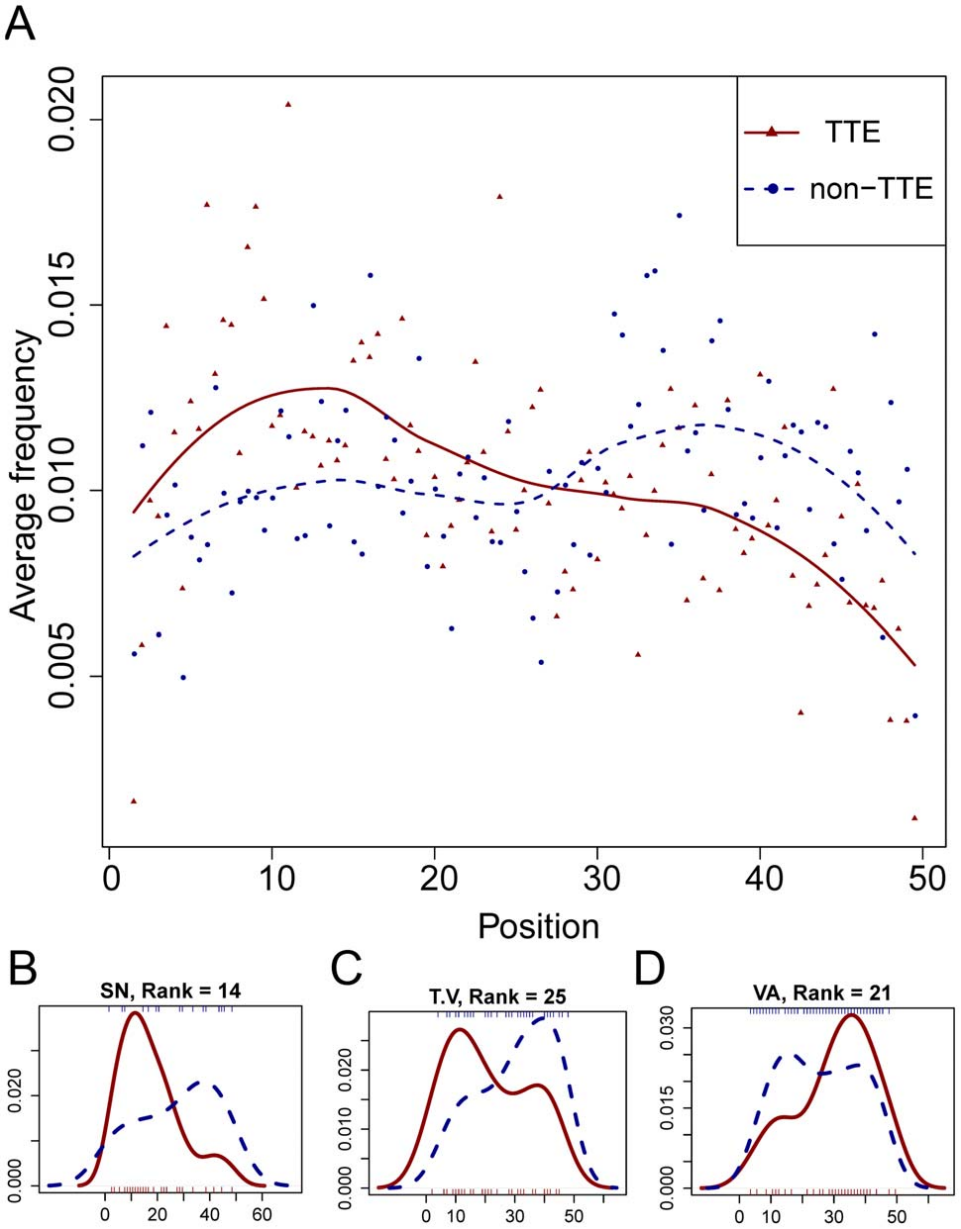
Sequence position distribution of *k*-spaced amino acid pairs. (**A**) Each point represents the overall frequency of the 50 most positively weighted amino acid pairs occurring at the N-terminal sequences from TTEs (red triangle) or non-TTEs (blue circle). Trend lines are drawn using *loess* smoothing for the points from TTEs (red) and non-TTEs (blue), respectively. (**B–D**) Position density distribution of pairs [SN], [T.V] and [VA] in TTEs (red solid line) and non-TTEs (blue dotted line). The horizontal axis in (**B–D**) is the same as in (**A**).

We analyzed the position distribution bias of the 50 most positively weighted amino acid pairs. Rather than being distributed promiscuously within the N-terminal sequences, these predictive amino acid pairs tend to occur at particular regions, which can facilitate the incorporation of some amino acid pairs into type-III secretion signal-related sequence motifs. As shown in Figure 4A, these 50 pairs tend to occur at the first 30 N-terminal residues of TTEs in comparison to the corresponding distributions in non-TTEs. The position distributions of individual pairs shows that, for most of these 50 pairs, there is a dominant density peak within the first 30 residues of the N-terminal sequences (Figure S2). In particular, the distribution distinction is very clear for some pairs related to serine. For example, the position density distribution of amino acid pair [SN] (rank = 14) shows a steep peak within the 10–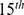 residues in TTEs, but there is only a flat peak near the 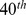 residue in non-TTEs (Figure 4B). The amino acid pair [S…P] (rank = 13) obviously appears within the 10–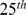 residues in TTEs, whereas it clusters within the 30–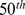 residues in non-TTEs (Figure S2). This clear distribution difference is also observed in some pairs containing no serine, such as [T.V] (rank = 25) (Figure 4C). We found a hydrophobic amino acid pair [VA] (rank = 21) that obviously tends to occur at the end of the first 50 residues in TTEs (Figure 4D). Recently, Costa et al. identified a sequence motif consisting of hydrophobic amino acids in the 30–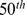 N-terminal residues of 15 TTEs, which is obligatory for completing type-III secretion [38]. Therefore, we believe that the 30–50^th^ residues should contain useful information about type-III secretion signals, and it is necessary to include these residues in our classification model, although only the first 30 residues were used in some existing predictors [14], [15].

### Comparison of BEAN and Four Existing Machine Learning-based Prediction Methods

**Figure 5:**
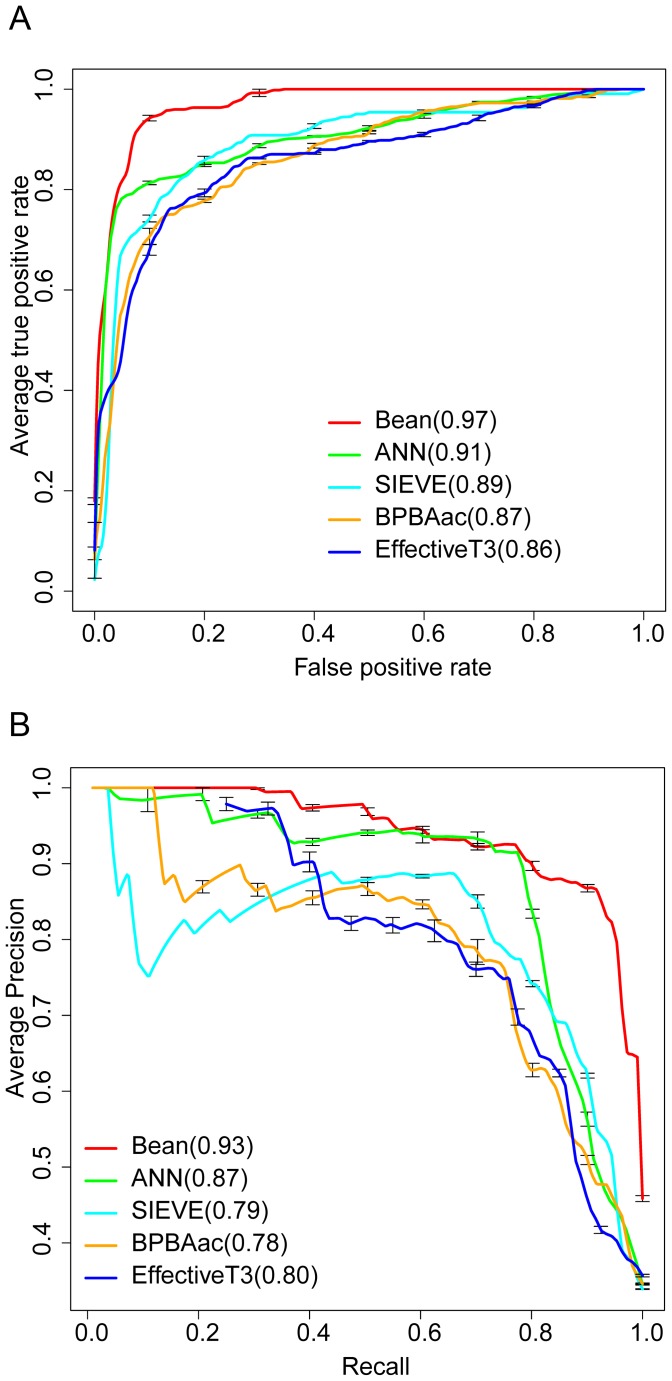
Comparison of different TTE predictors on the independent test set. (**A**) ROCs of five different methods. The values in the brackets are the average auROCs of each method. (**B**) Precision-recall curves of five different methods. Values in brackets are the average auPRCs of each method.

To facilitate the community, we have made BEAN freely available at http://protein.cau.edu.cn:8080/bean/. Note that the current predictor implemented in the BEAN webserver was trained on the whole dataset of Wang et al. (2011) [10]. We compared BEAN with four existing TTE predictors (i.e., EffectiveT3, SIEVE, ANN and BPBAac) based on an independent dataset (i.e., Data2). The EffectiveT3 and BPBAac software packages were downloaded from their websites and their performance on Data2 was tested in our local machine. Because stand-alone packages of ANN and SIEVE are not available, we submitted the sequences in Data2 directly to their webservers to obtain the prediction results. Note that only the EffectiveT3 model trained for both animal and plant pathogens was used for comparison. As shown in Figure 5, BEAN achieved a successful performance with an auROC value of 0.97 (auPRC = 0.93), which is considerably better than the other four methods.

We further investigated the performance of these five predictors on some special examples. Recently, 15 TTEs, which were not included in Data2 and only three of them were included in Data1, were characterized to contain a type-III secretion associated functional motif in the first 30∼50^th^ residues [38]. Since these 15 TTEs could not be successfully identified by some known machine learning methods [38], they are regarded as hard TTE samples. Interestingly, we found BEAN predicted 13 TTEs correctly when FPR was set to 5%. Using the same FPR control, ANN, SIEVE, BPBAac and EffectivT3 only correctly predicted 10, 8, 8 and 8 TTEs, respectively. We also focused on the prediction of 14 non-TTEs in Data2. These 14 proteins share high sequence identity with known TTEs, but experimental results have clearly shown that they can not be transported into host cells [39].Therefore, these 14 proteins should be regarded as hard non-TTE samples. Unfortunately, all of the five methods (BEAN, ANN, SIEVE, BPBAac and EffectivT3) can only correctly predict 7, 9, 7, 6 and 8, respectively. The poor prediction performance on these 14 non-TTEs indicates the current methods are still heavily dependent on the knowledge of known TTE sequences, which could limit these methods’ practical application to some extent. Furthermore, we observed that 12 non-TTEs were correctly predicted by at least one predictor, suggesting that these five predictors’ performances were complementary to some extent. We further used a majority vote strategy to integrate these five predictors, in which we took a sample as non-TTE (or TTE) if it was predicted as non-TTE (or TTE) in at least three predictors. Finally, the combined predictor successfully identified 10 non-TTEs. Although the accuracy improvement is limited in this case, the results showed that the combination of different predictors is a reasonable way to obtain better performance on hard samples.

Considering that existing predictors were generally developed using different datasets, performance comparison based on an independent test set is still subjective to some extent. For instance, we may argue that the improved performance of BEAN could be caused by a larger training set in comparison to some existing methods (e.g., EffectiveT3). We retrained BEAN using 92 non-redundant TTEs from the training data of EffectiveT3 and 184 randomly sampled non-TTEs from Wang et al.’s data [10], and tested this new classification model on Data2. In terms of auROC and auPRC, we still observed that BEAN can outperform the existing four methods, although its performance was slightly decreased (Figure S3). This result demonstrates that the improved performance of BEAN should be ascribed to the new methodology it adopts rather than the choice of the training dataset.

### Genome-wide TTE Identification in *R. solanacearum*

We conducted a genome-wide TTE identification in *R. solanacearum*. As one gram-negative bacterium, *R. solanacearum* can lead to bacterial wilt in tomato, banana and potato. The sequencing of the genome of *R. solanacearum* GMI1000 was completed in 2002 [40]. The protein sequences of *R. solanacearum* were downloaded from Uniprot, and the corresponding number of proteins is 4824. The prediction was performed only on those proteins containing more than 51 residues. Interestingly, 5 out of the 50 predicted TTEs with the highest prediction scores were experimentally validated and 24 predicted TTEs were annotated as putative TTEs in Uniprot (Table S2), implying that the performance of our prediction results should be generally good. It’s worth noting that known TTEs in *R. solanacearum* are not included in BEAN’s train dataset. Therefore, BEAN should be generally applicable to predict TTEs from newly sequenced pathogen genomes.

**Figure 6:**
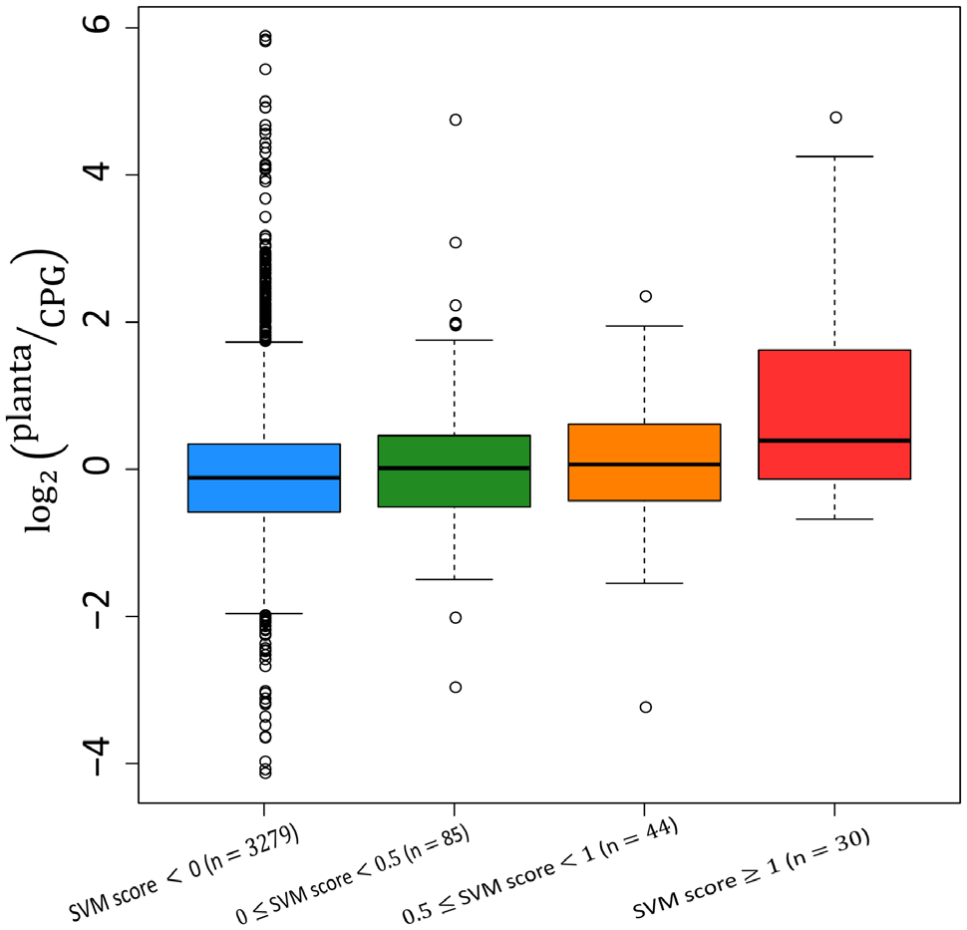
Gene differential expression distribution of prediction results. The vertical axis represents the fold changes of the gene expression level when *R. solanacearum* is cultured in tomato (planta) in comparison to the situation when *R. solanacearum* is cultured in rich medium (CPG). The number in the bracket is the gene number within this score interval. The statistically significant expression difference is observed between genes with SVM scores <0 and genes with SVM scores ≥1.0 (Mann-Whitney *U*-test, *p*-value <0.01).

To further provide an indirect assessment of our prediction results, we downloaded the microarray data of *R. solanacearum* from NCBI Gene Expression Omnibus (GEO), with the series number GSE33657. In this microarray experiment, the genome-wide mRNA expression level changes of *R. solanacearum* were measured when bacteria were cultured in rich medium CPG (Casamino acid-Peptone-Glucose) and tomato cell at 28°C. The limma package from Bioconductor (http://www.bioconductor.org/) was used to perform gene differential expression analysis. If a probe could not be mapped on any protein entry record from Uniprot, we ignored it when drawing the gene expression distribution. A Mann-Whitney *U*-test was used to conduct differential expression significance analysis between gene groups with different SVM output scores. Interestingly, we observed that genes with higher prediction scores tend to be upregulated when cultured in tomato (Figure 6). Therefore, the results of this investigation of gene expression difference partly validate the effectiveness of BEAN in identifying TTEs at the whole genome level.

Considering only a very small fraction of proteins in a genome are TTEs, we might underestimate the FPR of prediction results when we apply BEAN on a whole genome level. For each prediction result, therefore, we also provided a posterior probability at the genome level based on Bayes theorem (see Table S2, Text S1). The posterior probability can be used to further evaluate the reliability of a prediction result.

## Discussion

### Type-III Secretion Signals and Weakly Conserved Motifs

Although bacterial TTEs evolve at a high speed under the strong evolutionary selection stress from the host immune system, T3SS is relatively conserved [41] and different TTEs can be secreted by the same T3SS. Until now, the exact form of the Type-III secretion signal remains largely unknown. Although the hypothesis that Type-III secretion signals could be comprised of some sequence motifs has been proposed, previous studies could not successfully identify the sequence motifs from the MSA of type-III effectors, which may be ascribed to the sequence diversity of TTEs driven by the high evolutionary rate obscuring the form of sequence motifs in type-III secretion signals.

In this work, we developed BEAN based on the composition of *k*-spaced amino acid pairs. Because amino acid pairs can be regarded as the essential elements of sequence motifs, the high accuracy of BEAN partly confirmed the important role of sequence motifs in type-III secretion signals. We also note that the success of BEAN should be ascribed to the use of profile-based composition, which can effectively capture the evolutionary information of amino acid pairs from the homologues of TTEs, as the input for BEAN. This further suggested that Type-III secretion signal-related motifs should be weakly conserved, which can be exemplified by the following two examples related to computationally or experimentally identified sequence motifs. As reported in [15], Samudrala et al. identified two conserved sequence motifs from the first 30 residues of TTEs through statistical analysis. Interestingly, we found some amino acid pairs from these two motifs, such as [S.S], [SS] and [N…S], can also be found in our 50 most positively in Data2 and only three of them were included in Data1, were characterized to contain a type-III secretion associated f [LMIF…IV.IV.N] that was located in the 30–50^th^ N-terminal residues of 15 TTEs [38]. Because this motif has been proven to interact with chaperones involved in type-III secretion, it was named the conserved chaperone-binding domain (CCBD). Considering that the CCBD motif might be applicable in a limited number of TTEs, it is worth noting that we only observed one amino acid pair [V.N] from the CCBD motif appearing in our top 100 most positively weighted amino acid pairs (Rank = 60). It should be emphasized that there are only very limited motif cases found to be related with type-III secretion process, thus the association between potential type-III secretion signal related motifs and the predictive *k*-spaced amino acid pairs identified in this work need to be further tested with more experimentally verified TTEs.

### Composition of Type-III Secretion Signals

For some TTEs, the secretion signal may contain different types of motifs. The functional roles and position distribution could be different. The position distribution analysis of amino acid pairs shows that many of the 50 most important amino acid pairs we identified from TTEs tend to occur within the first 30 N-terminal residues (Figure 4), which have been proven to be enough for successful type-III secretion in some effectors [17], [18]. These informative N-terminal amino acid pairs primarily consisted of serine and other polar amino acids, suggesting that the secretion signal in this region should contain motifs enriched in serine or other polar residues.

We also note that some hydrophobic amino acid pairs (e.g., [VA]) are prone to appear in the 30–45^th^ residues, suggesting that hydrophobic short motif-related secretion signals should exist in this region. Generally, hydrophobic short motifs can mediate protein-protein interactions [24]. Similar to the CCBD motif, these hydrophobic short motifs in the 30–50^th^ N-terminal residues may also serve as chaperone binding motifs. Although chaperone binding motifs are obligatory for the successful type-III secretion process of some TTEs, their functional roles in the recognition between TTEs and type-III secretion systems have not been fully deciphered [41]. Recently, Galán and co-workers demonstrated that some TTEs from *Salmonella enterica* serovar Typhimurium can be sorted through customized chaperones before secretion to determine the order of passing T3SS [42]. Combining these experimental discoveries and our analysis, we argue that type-III secretion might contain a composite signal, including the first level signal within the front of N-terminal sequences and the second level signal within the tail of N-terminal sequences. The first level signal consists of polar amino acids, which are a common feature for most TTEs. The second level signal that contains hydrophobic motifs might be customized for different TTEs to interact with their specific chaperones. It is possible that type-III secretion is an integrated result of a multiple-step recognition process.

### Future Work

To the best of our knowledge, all of the current machine learning-based TTE predictors (including BEAN) only consider two classes of proteins (i.e., TTEs and non-TTEs) and do not classify TTEs into different sub-types. This simple binary classification might be not very reasonable provided that the mechanisms of type-III secretion signal recognition are slightly different in different TTEs. Thanks to the research community’s efforts, experimentally verified TTEs are increasing rapidly. A comprehensive and hierarchical TTE classification system will be highly desirable. Undoubtedly, this will be very helpful for unveiling the secret of TTE secretion signals and developing customized prediction models for each TTE type.

## Supporting Information

Figure S1 Performance of each type of *k*-spaced amino acid pairs. We exclusively used each type of *k*-spaced amino acid pairs (i.e., *k* = 0, 1, 2 or 3 was individually used) to train the corresponding predictive model and we called the resulting four SVM models as exclusive-CKSAAP. The dipeptide encoding can be regarded as exclusive-CKSAAP (*k* = 0). The values in brackets are the auROCs of different SVM models.
(TIFF)

Figure S2 Position density distribution of the 50 most predictive *k*-spaced amino acid pairs. Red lines stands for amino acid pairs in TTEs and blue lines stands for amino acid pairs in non-TTEs. The horizontal and vertical axes are the same as in Figure 4.
(PDF)

Figure S3 Performance of BEAN on Data2 when BEAN’s classification model was retrained with EffectiveT3 dataset. **(A)** ROCs of five different methods. The values in the brackets are the average auROCs of each method (or classification model). **(B)** Precision-recall curves of five different methods. Values in brackets are the average auPRCs of each method (or classification model).
(TIFF)

Table S1 The performance of BEAN using different negative samples.
(DOC)

Table S2 BEAN’s top 50 prediction results on the whole genome of *Ralstonia solanacearum* GMI1000.
(DOC)

Text S1 Estimation of a prediction result’s posterior probability at the genome level.
(DOC)

Dataset S1 Protein sequences in Data1. This dataset includes 462 sequences from Wang et al.’s data and four groups of non-redundant non-TTEs sequences (308 sequences for each group) randomly sampled from gram-negative pathogenic bacterial proteomes.
(ZIP)

Dataset S2 Protein sequences in Data2. This dataset includes 109 newly identified TTEs and 14 experimentally validated non-TTEs collected manually from literature published after January 2011, and five groups of non-TTEs sequences (200 sequences for each group) randomly sampled from gram-negative pathogenic bacterial proteomes.
(ZIP)
