# Supplementary figures and images for: Using Weakly Conserved Motifs Hidden in Secretion Signals to Identify Type-III Effectors from Bacterial Pathogen Genomes
Source: PLoS One. 2013 Feb 20;8(2):e56632. doi: 10.1371/journal.pone.0056632 (PMC3577856; doi:10.1371/journal.pone.0056632)

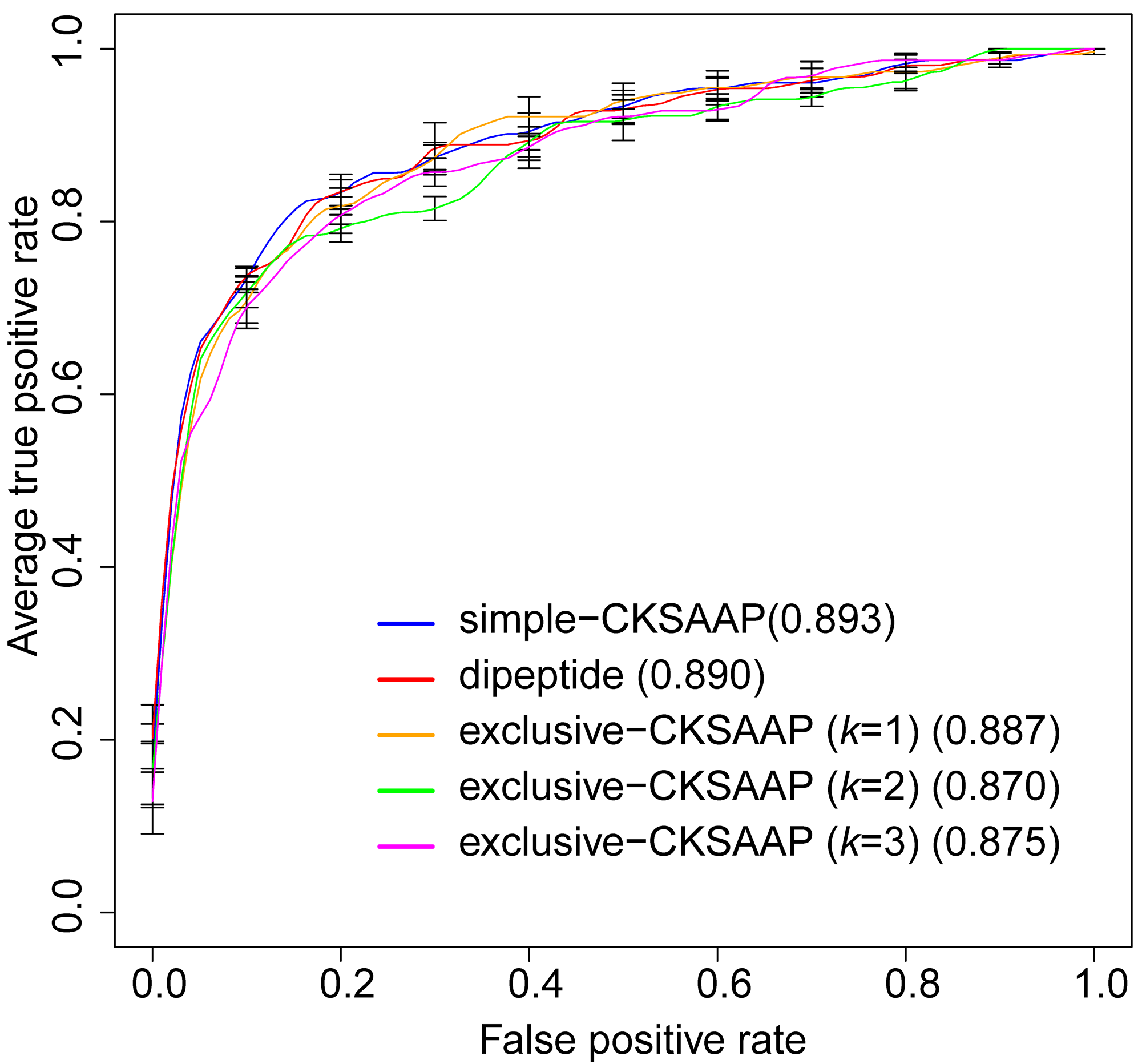

Supplement: Figure S1 — Performance of each type of k-spaced amino acid pairs. We exclusively used each type of k-spaced amino acid pairs (i.e., k = 0, 1, 2 or 3 was individually used) to train the corresponding predictive model and we called the resulting four SVM models as exclusive-CKSAAP. The dipeptide encoding can be regarded as exclusive-CKSAAP (k = 0). The values in brackets are the auROCs of different SVM models. (TIFF) [file pone.0056632.s001.tif]

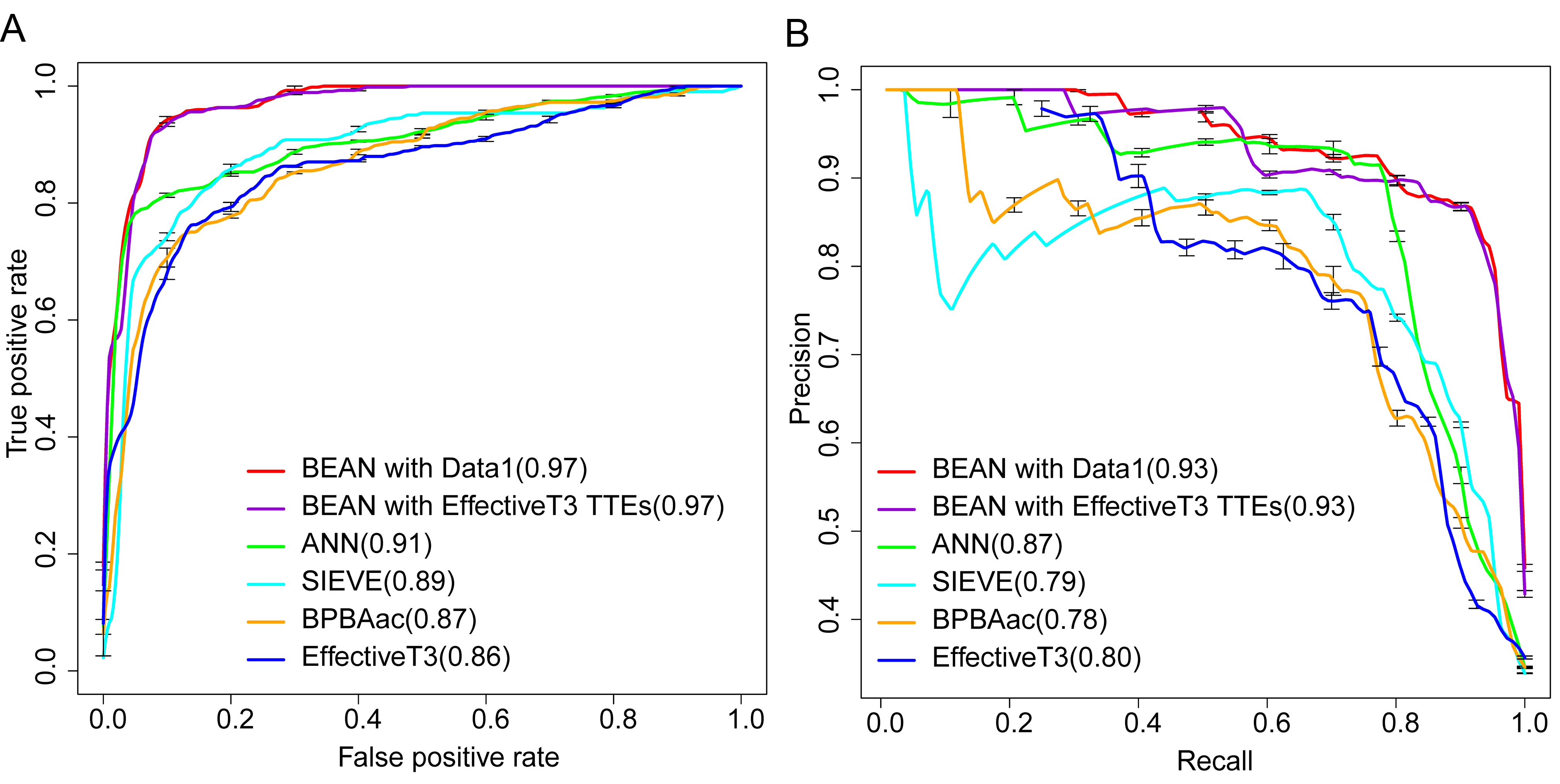

Supplement: Figure S3 — Performance of BEAN on Data2 when BEAN’s classification model was retrained with EffectiveT3 dataset. (A) ROCs of five different methods. The values in the brackets are the average auROCs of each method (or classification model). (B) Precision-recall curves of five different methods. Values in brackets are the average auPRCs of each method (or classification model). (TIFF) [file pone.0056632.s003.tif]
