# Supplementary material for: Using Weakly Conserved Motifs Hidden in Secretion Signals to Identify Type-III Effectors from Bacterial Pathogen Genomes
Source: PLoS One. 2013 Feb 20;8(2):e56632. doi: 10.1371/journal.pone.0056632 (PMC3577856; doi:10.1371/journal.pone.0056632)
Supplement: Figure S2 — Position density distribution of the 50 most predictive k-spaced amino acid pairs. Red lines stands for amino acid pairs in TTEs and blue lines stands for amino acid pairs in non-TTEs. The horizontal and vertical axes are the same as in Figure 4. (PDF) [file pone.0056632.s002.pdf]

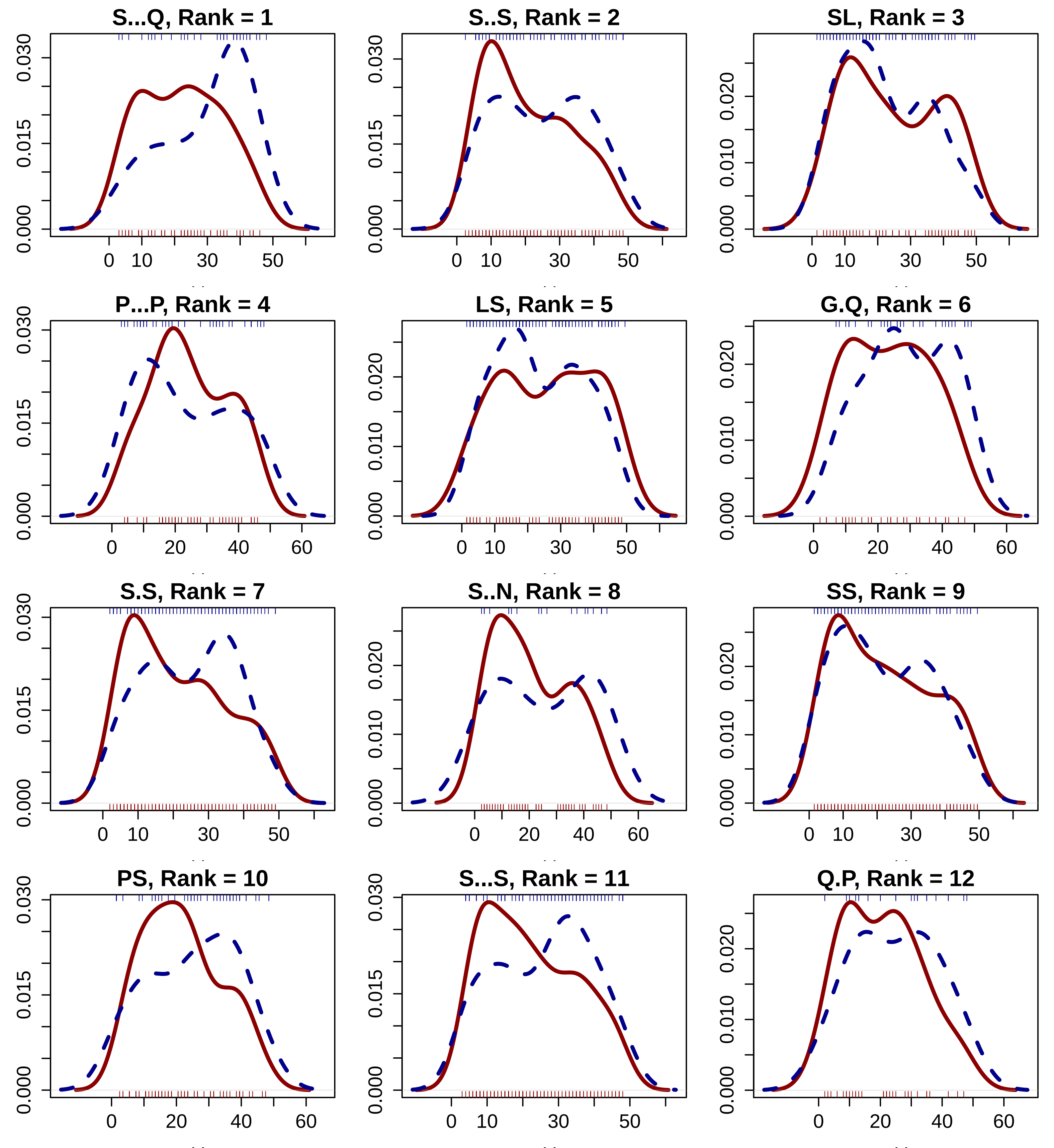
S...Q, Rank = 1
S..S, Rank = 2
SL, Rank = 3
P...P, Rank = 4
LS, Rank = 5
G.Q, Rank = 6
S.S, Rank = 7
S..N, Rank = 8
SS, Rank = 9
PS, Rank = 10
S...S, Rank = 11
Q.P, Rank = 12
0.000
0.015
0.030
0.010
0.020
0
10
20
30
40
50
60

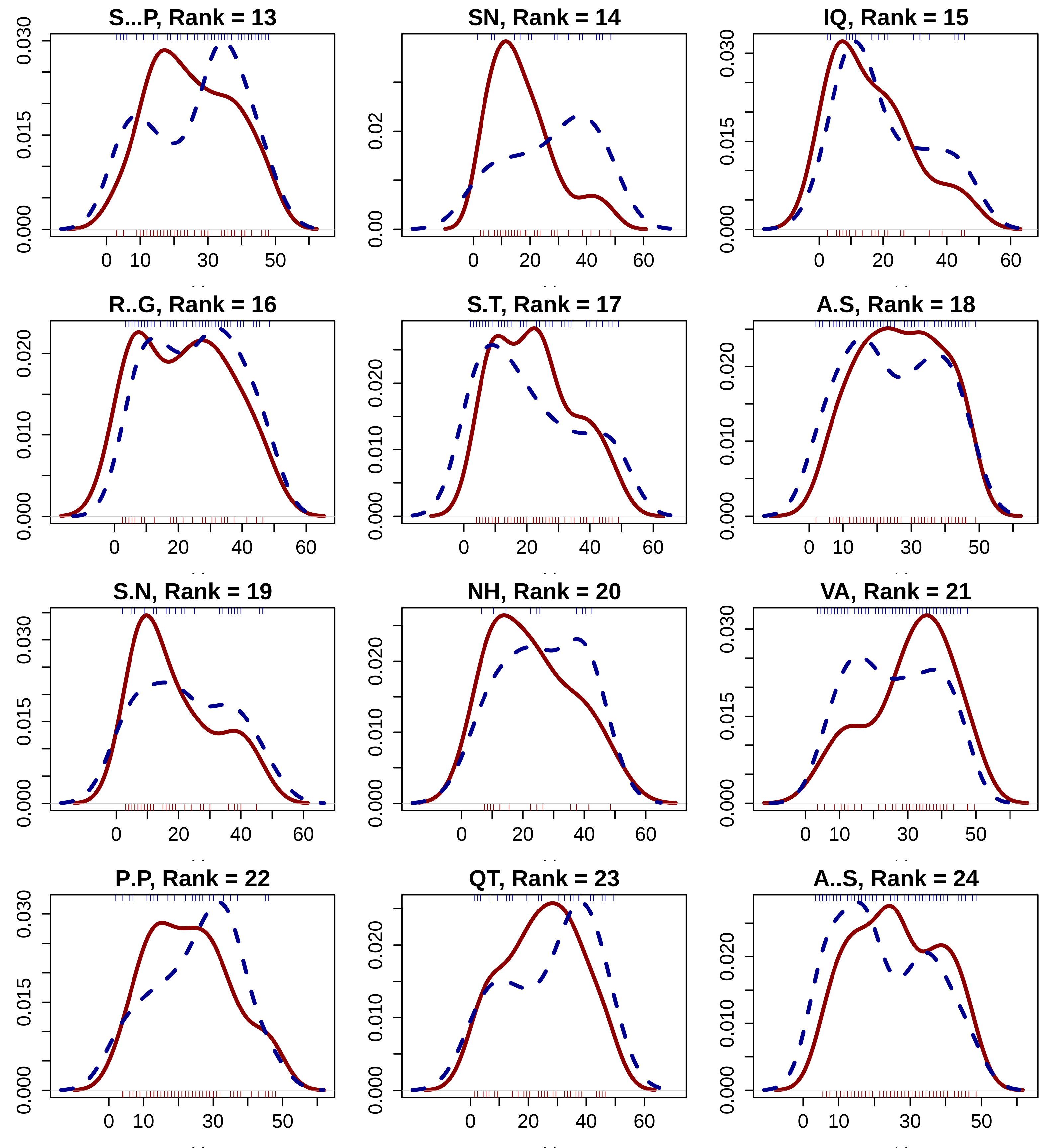
S...P, Rank = 13
0.000
0.015
0.030
0
10
30
50
SN, Rank = 14
0.00
0.02
0
20
40
60
IQ, Rank = 15
0.000
0.015
0.030
0
20
40
60
R..G, Rank = 16
0.000
0.010
0.020
0
20
40
60
S.T, Rank = 17
0.000
0.010
0.020
0
20
40
60
A.S, Rank = 18
0.000
0.010
0.020
0
10
30
50
S.N, Rank = 19
0.000
0.015
0.030
0
20
40
60
NH, Rank = 20
0.000
0.010
0.020
0
20
40
60
VA, Rank = 21
0.000
0.015
0.030
0
10
30
50
P.P, Rank = 22
0.000
0.015
0.030
0
10
30
50
QT, Rank = 23
0.000
0.010
0.020
0
20
40
60
A..S, Rank = 24
0.000
0.010
0.020
0
10
30
50

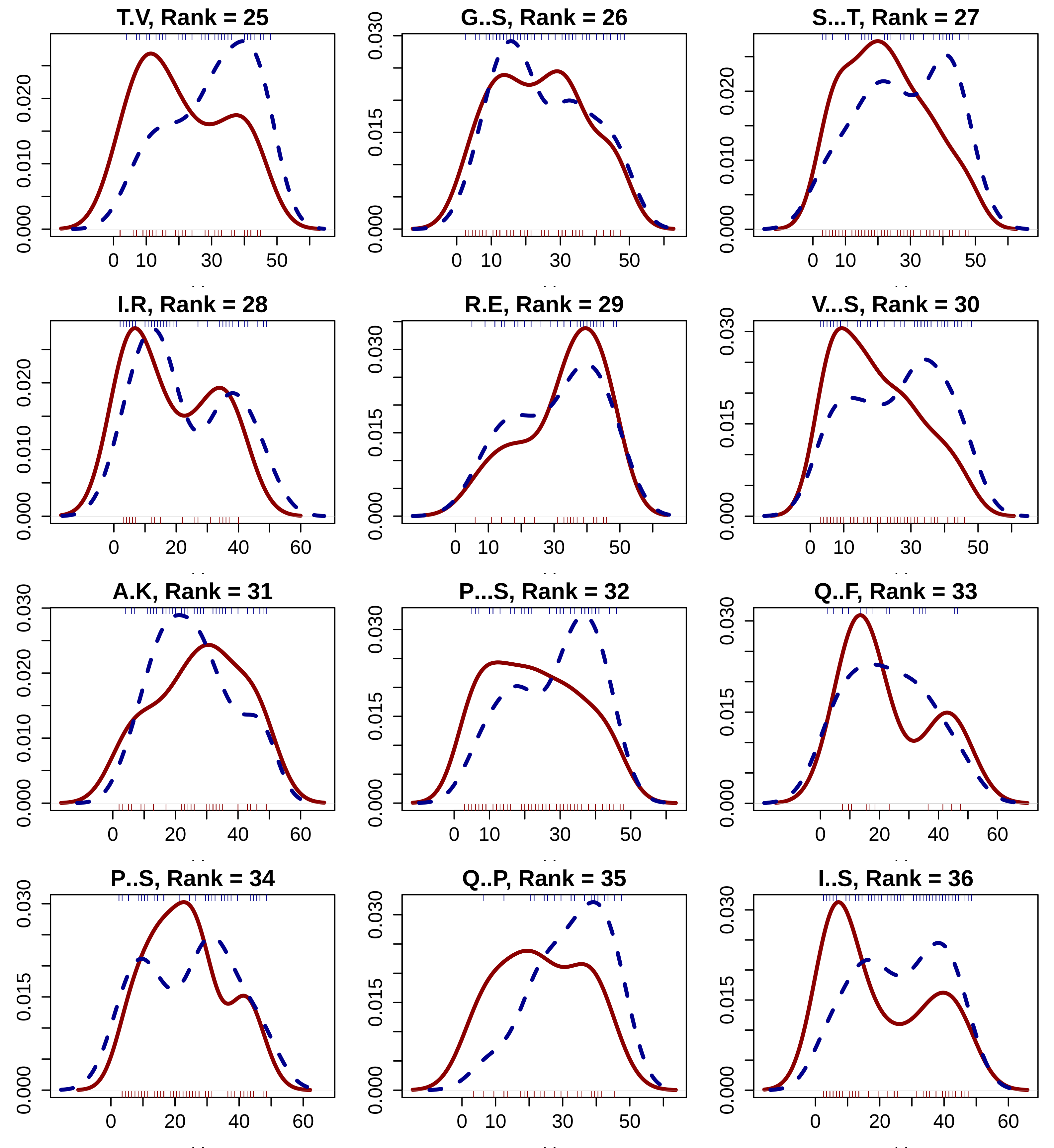
T.V, Rank = 25
G..S, Rank = 26
S...T, Rank = 27
I.R, Rank = 28
R.E, Rank = 29
V...S, Rank = 30
A.K, Rank = 31
P...S, Rank = 32
Q..F, Rank = 33
P..S, Rank = 34
Q..P, Rank = 35
I..S, Rank = 36
0.000
0.010
0.020
0.015
0.030
0
10
20
30
40
50
60

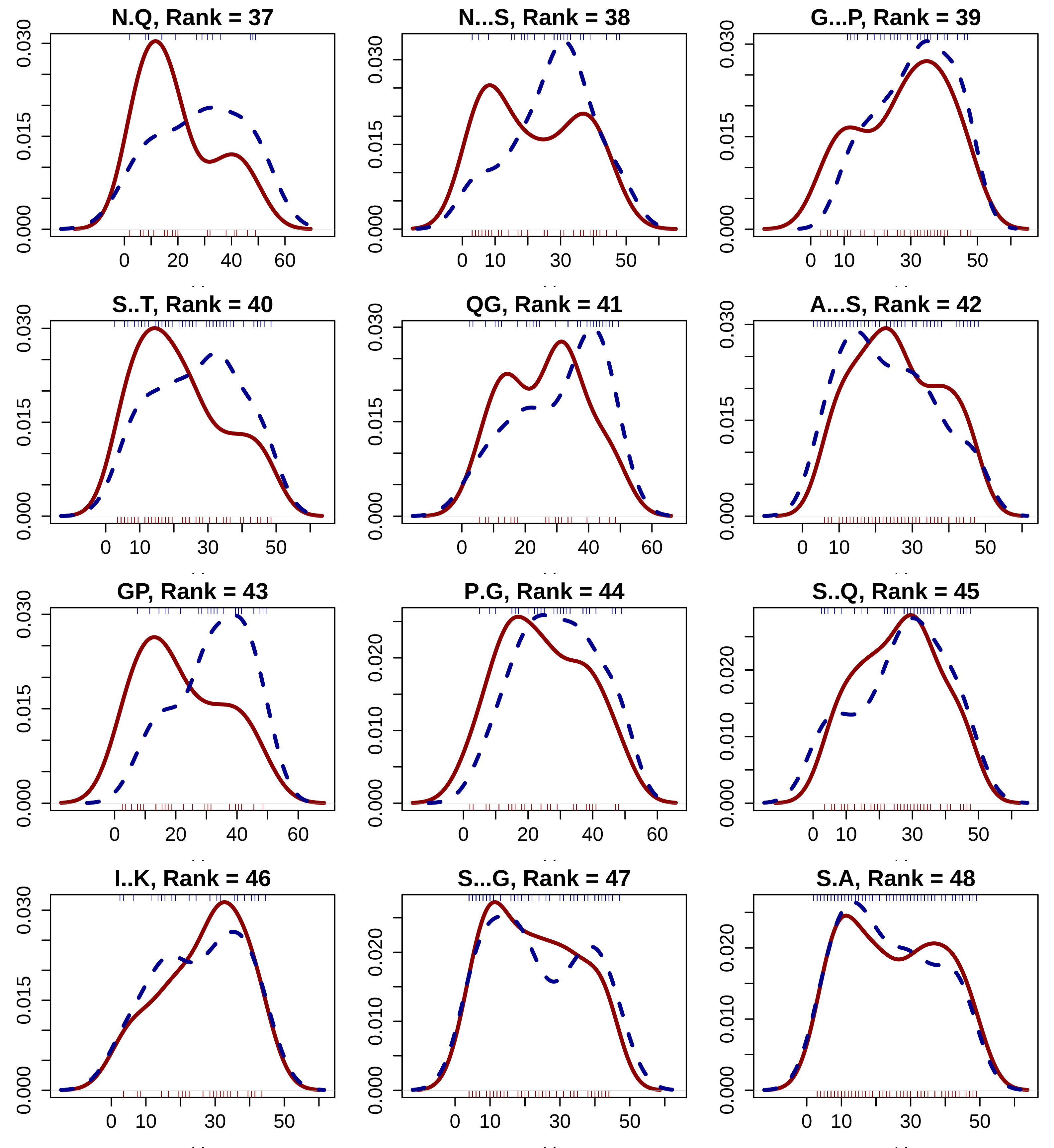
N.Q, Rank = 37
N...S, Rank = 38
G...P, Rank = 39
S..T, Rank = 40
QG, Rank = 41
A...S, Rank = 42
GP, Rank = 43
P.G, Rank = 44
S..Q, Rank = 45
I..K, Rank = 46
S...G, Rank = 47
S.A, Rank = 48
0.000
0.015
0.030
0.010
0.020
0
10
20
30
40
50
60

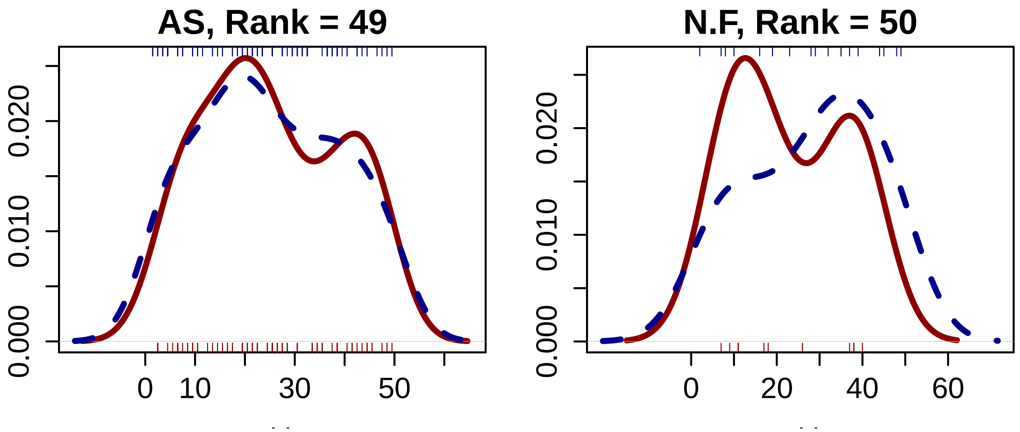
AS, Rank = 49
N.F, Rank = 50
0.000
0.010
0.020
0
10
30
50
20
40
60
