# Supplementary material for: Using Weakly Conserved Motifs Hidden in Secretion Signals to Identify Type-III Effectors from Bacterial Pathogen Genomes
Source: PLoS One. 2013 Feb 20;8(2):e56632. doi: 10.1371/journal.pone.0056632 (PMC3577856; doi:10.1371/journal.pone.0056632)
Supplement: Table S1 — The performance of BEAN using different negative samples. (DOC) [file pone.0056632.s004.doc]

**Table S1.** The performance of BEAN using different negative samples..

| **Dataset** | **Accuracy** | **Sensitivity** | **Specificity** | **MCC** |
| --- | --- | --- | --- | --- |
| Wang et al's | 0.9 | 0.78 | 0.96 | 0.78 |
| nr_neg_2 | 0.92 | 0.79 | 0.98 | 0.81 |
| nr_neg_3 | 0.91 | 0.79 | 0.97 | 0.8 |
| nr_neg_4 | 0.92 | 0.79 | 0.98 | 0.81 |
| nr_neg_5 | 0.93 | 0.82 | 0.99 | 0.84 |
