# Supplementary material for: Using Weakly Conserved Motifs Hidden in Secretion Signals to Identify Type-III Effectors from Bacterial Pathogen Genomes
Source: PLoS One. 2013 Feb 20;8(2):e56632. doi: 10.1371/journal.pone.0056632 (PMC3577856; doi:10.1371/journal.pone.0056632)
Supplement: Table S2 — BEAN’s top 50 prediction results on the whole genome of Ralstonia solanacearum GMI1000. (DOC) [file pone.0056632.s005.doc]

**Table S2. BEAN’s top 50 prediction results on the whole genome of *Ralstonia solanacearum* GMI1000.^a^**

| **Uniprot AC** | **Score** | **Prob.** | **Uniprot Annotation** |
| --- | --- | --- | --- |
| **Q8XUU0_RALSO** | 2.76693 | 1 | Putative serine-rich protein |
| **Q8XQA2_RALSO** | 2.52655 | 1 | Putative type III effector protein |
| **POPA_RALSO** | 1.97346 | 1 | Acts as a specific hypersensitive response (HR) elicitor. Has activity on tobacco (non-host plant) and petunia but is without activity on tomato (host plant) |
| **Q8XQI6_RALSO** | 1.87362 | 1 | Putative type III effector protein |
| **Q8XVM6_RALSO** | 1.83542 | 1 | Putative transmembrane ribonucleoside reductase (Small chain) oxidoreductase protein |
| **Q8XT13_RALSO** | 1.7663 | 1 | Probable avrpphd family type III effector protein |
| **Q1MTJ7_RALSO** | 1.75952 | 1 | Probable n-terminal part of a truncated yopp/avrrvx-related protein |
| **Q8XTA1_RALSO** | 1.75212 | 1 | Putative type III effector protein |
| **Q8XSD2_RALSO** | 1.70241 | 1 | Putative uncharacterized protein |
| **Q8XZC6_RALSO** | 1.6993 | 1 | Putative type III effector protein |
| **Q8XZP6_RALSO** | 1.68994 | 1 | Putative type III effector protein |
| **Q8XRI6_RALSO** | 1.68886 | 1 | Putative type III effector protein |
| **Q8XPG7_RALSO** | 1.68375 | 1 | Hypothetical lipoprotein transmembrane |
| **Q8XYZ7_RALSO** | 1.61154 | 1 | Putative d-alanyl-d-alanine-endopeptidase (Penicillin-binding protein) |
| **Q8XRQ4_RALSO** | 1.58058 | 1 | Putative transmembrane protein |
| **Q8Y2T7_RALSO** | 1.50055 | 1 | Type III effector ripb protein |
| **Q8XR37_RALSO** | 1.47248 | 1 | Putative type III effector protein |
| **Q8XQL0_RALSO** | 1.44799 | 1 | Putative type III effector protein |
| **POPB_RALSO** | 1.42134 | 1 | Probably involved in host-pathogen interactions |
| **Q8XPQ6_RALSO** | 1.41349 | 1 | Putative type III effector protein |
| **Q8Y125_RALSO** | 1.40213 | 1 | Type III effector protein popp2 |
| **Q8XUL4_RALSO** | 1.36508 | 1 | Putative type III effector protein |
| **Q8XQ11_RALSO** | 1.32478 | 1 | Putative type III effector protein |
| **Q8Y2L4_RALSO** | 1.32476 | 1 | Putative type III effector protein |
| **Q8XRI4_RALSO** | 1.29095 | 1 | Probable awr type III effector family protein |
| **Q8Y167_RALSO** | 1.2902 | 1 | Putative uncharacterized protein |
| **Q8XQT8_RALSO** | 1.28544 | 1 | Putative type III effector protein |
| **Q8Y116_RALSO** | 1.27778 | 1 | Putative uncharacterized protein |
| **Q8XXV9_RALSO** | 1.26786 | 1 | Probable signal peptide protein |
| **Q8XXT5_RALSO** | 1.25882 | 1 | Putative uncharacterized protein |
| **Q8XS95_RALSO** | 1.24135 | 1 | Putative uncharacterized protein |
| **Q8XQ26_RALSO** | 1.24012 | 1 | Putative type III effector protein |
| **Q8XQ71_RALSO** | 1.2401 | 1 | Putative uncharacterized protein |
| **Q8Y376_RALSO** | 1.23373 | 1 | Putative uncharacterized protein |
| **Q8XQF0_RALSO** | 1.22234 | 1 | Putative type III effector protein |
| **Q8XTL7_RALSO** | 1.21323 | 1 | Putative uncharacterized protein |
| **Q8XXH5_RALSO** | 1.19187 | 1 | Awr type III effector family protein |
| **Q8XZ55_RALSO** | 1.16128 | 1 | Putative class II aldolase/adducin, n-terminal; protein |
| **Q8XQI9_RALSO** | 1.15322 | 0.4 | Putative type III effector protein |
| **Q8Y2V5_RALSO** | 1.13975 | 0.407186 | Probable pseudogene (Type III effector protein, avrpm1 homologue) |
| **Q8XPJ7_RALSO** | 1.13073 | 0.407186 | Putative uncharacterized protein |
| **Q8Y166_RALSO** | 1.10908 | 0.407186 | Putative type III effector protein |
| **Q8XUH6_RALSO** | 1.10025 | 0.414201 | Type III effector protein ript |
| **Q8XXA1_RALSO** | 1.09869 | 0.414201 | Sigma factors are initiation factors that promote the attachment of RNA polymerase to specific initiation sites and are then released |
| **Q8XPZ2_RALSO** | 1.08629 | 0.414201 | Putative uncharacterized protein |
| **Q8XPH0_RALSO** | 1.07686 | 0.414201 | Hypothetical signal peptide protein |
| **Q8XUL3_RALSO** | 1.06535 | 0.421053 | Probable transmembrane protein |
| **Q8Y3D9_RALSO** | 1.0628 | 0.427746 | Putative type III effector protein |
| **Q8XVQ5_RALSO** | 1.06109 | 0.427746 | Probable harpin protein with pectate lyase domain |
| **Q8Y0Z8_RALSO** | 1.05995 | 0.427746 | Putative type III effector protein |

^a^Uniprot AC is protein accession number in Uniprot database. Score is raw SVM prediction score of Bean. Prob. is Baysian posterior probability of the corresponding prediction result (The detailed calculation procedures of Prob. is available at Text S1).
