# Supplementary material for: Using Weakly Conserved Motifs Hidden in Secretion Signals to Identify Type-III Effectors from Bacterial Pathogen Genomes
Source: PLoS One. 2013 Feb 20;8(2):e56632. doi: 10.1371/journal.pone.0056632 (PMC3577856; doi:10.1371/journal.pone.0056632)
Supplement: Text S1 — Estimation of a prediction result’s posterior probability at the genome level. (DOC) [file pone.0056632.s006.doc]

**Estimation of a prediction result’s posterior probability at the genome level**

We computed posterior probability of each prediction result using Bayes theorem. The computing process could be described as follows:

Firstly, we presumed 1% proteins in a gram-negative pathogenic bacterium’s proteome were TTEs. The priori probability (*P_priori_*) is deduced from two well-studied pathogens *Salmonella enterica* serovar Typhimurium LT2 and *Pseudomonas syringae* DC3000. Both of the two proteomes contain around 5000 proteins, and approximately 40 and 28 effectors have been identified respectively[1]. Secondly, each TTE and non-TTE in Wang et al. (2011) data was predicted by Bean to assign a SVM prediction score. We took these 462 scores with known class labels as benchmark dataset ScoreRef. Thirdly, for a new prediction result, we took its SVM output score as a cutoff and used it to estimate true positive rate (TPR), false positive rate (FPR), true negative rate (TNR) and false negative rate (FNR) of ScoreRef. Posterior probability of a result with SVM output score (*S_raw_*) could be calculated:

if

else

where$P_{priori}=0.01$is the priori probability of TTEs’ occurrence in one pathogenic bacterial genome. *P_posteriori_*(i.e. Prob. in Table S3) could be seen as a support degree of a prediction result in the whole genome.

**References**

1. Sato Y, Takaya A, Yamamoto T (2011) Meta-analytic approach to the accurate prediction of secreted virulence effectors in gram-negative bacteria. BMC Bioinformatics 12: 442.

2. Wang Y, Zhang Q, Sun M-a, Guo D (2011) High-accuracy prediction of bacterial type III secreted effectors based on position-specific amino acid composition profiles. Bioinformatics 27: 777-784.
